# Supplementary material for: Genomic and functional determinants of host spectrum in Group B Streptococcus
Source: PLoS Pathog. 2024 Aug 12;20(8):e1012400. doi: 10.1371/journal.ppat.1012400 (PMC11341095; doi:10.1371/journal.ppat.1012400)
Supplement: Attachment — Submitted filename: Resubmission_Letter_Plos_Pathogens.pdf [file ppat.1012400.s006.pdf]

INSTITUT PASTEUR
Global Health

Paris, July 4th, 2024

Dear Editors of PLOS Pathogens,

Thank you for considering our manuscript "Genomic and functional determinants of host spectrum in Group B *Streptococcus*" for publication in PLOS Pathogens, and for sending us the reviewers' feedback. We have addressed all feedback thoroughly (as detailed in the response to reviewers) and revised our manuscript based on the reviewers' comments.

Regarding reviewer #1, we found their feedback quite general and sometimes unclear, lacking specific input on how to improve the manuscript. Notably, they did not suggest any modifications in parts II (major issues) and III (minor issues). Despite this, we have attempted to address their comments in part I (summary) as thoroughly as possible. We clarified our rationale for not selecting the Lac.2 operon and the *scpB* transposon for *in vivo* experiments, explaining the extensive existing literature supporting their roles in GBS infections thanks to both *in vitro* and *in vivo* data in cattle and humans, respectively.

For reviewer #2, we have incorporated all their constructive suggestions. We defined 'MGE' at first mention, explained our selection criteria for host generalists versus host specialists, reorganized Fig. 2B for better readability, and clarified the reference to SL61. Additionally, we have made changes to the caption of Fig. 4A, explaining why the smaller peak of human-associated unitigs in the Manhattan plot was not annotated in the figure. We also ensured that the clarity of supplemental data citations was improved, notably for the Supplementary Tables S1-S4.

Please find our detailed answers to reviewers below.

In addition, as per your request:

- We have updated the manuscript template with the LaTex one from PLOS Pathogens (uploaded in two copies, one with highlights on the modified text and a clean version), and we have uploaded the corresponding tex file
- Figures have been provided in tiff format
- We have checked and confirm the author contributions based on CRediT
- We updated the Financial Disclosure statement based on PLOS Pathogens guidelines, to uniquely include the funding bodies that specifically supported this project and related stipends
- We integrated an Author Summary to make our findings more accessible to a larger audience
- We have removed the "data not shown" statement and we have integrated further analyses for the *Streptococcus iniae* findings (lines 264-266 and in the Supplementary appendix Fig. C.3)
- We added a list of legends for the supplementary files
- We amended the Ethical statement to the following: "Animal experiments were in compliance with the ARRIVE guidelines and were carried out out in accordance with the U.K. Animals (Scientific Procedures) Act, 1986 and associated guidelines, and with the EU Directive 2010/63/EU for animal experiments. Challenge studies were conducted by a contract research organisation specialized in aquatic animal health (Ictyopharma, France). Studies were performed to Good Laboratory Practice standards, approved by an internal Ethics Committee and the French Ministry of Research, and audited by an independent Quality Assurance partner."
- With regards to potential copyright issues with Figure 1, we would like to reassure you that the host silhouettes were downloaded from a website that allows reuse of the images under Creative Commons licences, as stated in their main webpage: https://www.phylopic.org/

We are confident that our revised manuscript addresses all reviewers' comments and we trust it will satisfy the PLOS Pathogens Editorial Board.

INSTITUT PASTEUR
Global Health

Thank you very much for considering our revised submission.

Sincerely,

Chiara Crestani
DVM, PhD
Global Health Department
Institut Pasteur Paris
chiara.crestani@pasteur.fr

Ruth Zadoks
DVM, MSc, MRes, PhD, Professor
Sydney School of Veterinary Science
University of Sydney
ruth.zadoks@sydney.edu.au

Reviewer's Responses to Questions

Part I - Summary
Please use this section to discuss strengths/weaknesses of study, novelty/significance, general execution and scholarship.

Reviewer #1: Chiara Crestani and colleagues described the global GBS population comprises host-generalist, host-adapted and host-restricted sublineages, as they named, through bioinformatics analysis by downloading the whole genome sequence data from public repositories or self-generated. This is an interesting idea.

However, the manuscript seemed to lack novelty. The evidence in support of the authors' statement is insufficient. Furthermore, we think the manuscript was not well-organized.
For example, In line 2.5. In vivo assessment of the role of Locus 3 in fish infection.
Why the Lac.2 operon (GBS in cattle) and the scpB transposon (GBS in humans) were not selected for in-vivo experiment. The author should explain this in the introduction.

We want to thank the reviewer for dedicating their time to evaluating our manuscript. However, we found the reviewer's feedback quite general, unclear in its criticism and lacking specific input on how to ameliorate the manuscript (for instance, no suggestions were provided in parts II and III).

We respectfully disagree with their appraisal that our study lacks novelty. We believe our work contributes novel findings, which is also highlighted by reviewer #2, as it: i) represents the first GWAS study investigating genetic determinants of host adaptation in Group B Streptococcus (GBS), and points to a pivotal role of a few genetic determinants of host-adaptation (scpB, Lac.2 and Locus 3); ii) significantly adds to our understanding and knowledge of the dynamics of host-switches and evolution of GBS which appear to be driven by said genetic determinants, and which are particularly relevant e.g., in the case of the hypervirulent clone CG283 (section 3.6.1); iii) demonstrates for the first time the critical role and function of the Locus 3 gene cluster in fish infections, with important repercussions in the expansion of hypervirulent GBS in this host group which now represents CG283 major reservoir, as supported by our traits analysis on Bayesian time-scaled phylogenies (section 3.6.1).

We are unable to respond to the reviewer's comment "The evidence in support of the authors' statement is insufficient". Our paper contains several analyses, and therefore multiple lines of evidence and statements based upon said evidence; as such, it is unclear to the authors to which evidence, and to which statement the reviewer is referring to.

Could the reviewer also kindly make suggestions on how this paper could be better organised, or specify which parts do not appear well-organised to them? The manuscript has been formatted following PLOS Pathogens guidelines, with an introduction, materials and methods (with subsections for each relevant experiment or analysis), results and discussion, and conclusions section. In particular, the authors made sure to organise the results and discussion section in sub-sections, each dedicated to a different analysis/experiment, and each proceeded by a clear title conveying the main result(s) of that analysis/experiment.

Finally, the authors did not carry out further in vivo experiments for scpB and Lac.2 as extensive evidence already exists in the literature for the functional role of these genetic determinants in human and bovine infections, respectively. With regards to scpB, it has been shown in both in vitro and in vivo murine models how scpB (encoding a c5a-peptidase, a surface-associated serine protease) (information added in line 29) inactivates human, but not murine, C5a, which is crucial for acute neutrophil response to GBS (Bohnsack JF et al.,

1997), by cleaving the C5a complement component (Bohnsack JF et al., 1991), how it promotes invasion by binding to human fibronectin (Cheng Q et al., 2002, Beckmann C et al., 2002) and how it is selectively activated by human serum but not by bovine serum (Glech-Theurer U et al., 2009), underlining its importance as virulence factor in GBS human infections (see lines 228-231). With regards to Lac.2 in GBS infections of bovines, which are known to be restricted to the mammary gland, the role of the genes comprised in this element has been previously shown by both with in-vitro lactose fermentation (Richards et al., 2013, Lyhs et al., 2016) and by transcriptomic approaches (Richards et al., 2013). Fermentation of lactose via lactose operons is known to promote growth in lactose-rich environments such as the bovine udder not only in other pathogenic streptococci (including GBS, S. uberis, S. dysgalactiae subsp. dysgalactiae) (Richards et al., 2011, Richards et al., 2013, but also in Gram-negative bacteria such as Klebsiella pneumoniae (Holt et al., 2015) (see lines 244, 248-252) and E. coli (Pinto C., 2021), and it is crucial for milk acidification processes needed for cheese production with e.g. Streptococcus thermophilus (Selle K et al., 2019, Xiong ZQ et al., 2019) and Lactobacillus casei (Alpert CA and Siebers U, 1997). As the authors strive to operate within a 3Rs framework (Replacement, Reduction, Refinement) to prioritize animal welfare, and as we considered the available evidence for the functions of both scpB and Lac.2 extensive and convincing, we deemed not necessary to pursue further in-vivo experiments in murine or bovine models. This was not the case for Locus 3, which is why we decided to proceed with in-vivo challenge experiments in fish after mutagenesis experiments (this explanation has been added in lines 272-275).

Reviewer #2: In this work, Crestani et al., analyze a robust set of 1,254 high-quality GBS genomes to identify potential drivers of host specificity. They identify three functional groups that describe host specificity – host generalists, host-adapted, and host restricted and assign GBS clonal groups (CGs) within these classes to the host species. They then identify genetic drivers that contribute to host-adaptation reiterating the association of scpB acquisition with human interactions and the Lac.2 operon with cattle. Interestingly they identify a novel genetic locus they termed Locus 3 that is associated with adaptation to fish hosts, and they show loss of Locus 3 significantly decreases GBS virulence in two distinct strain backgrounds and animal models. The findings presented confirm previous claims about scpB and Lac.2 while also contributing novel findings for GBS evolution in the context of fish hosts.

We thank the reviewer for the appreciation of our work and for highlighting the robustness of our analyses, as well as the novelty represented by the vast GWAS analysis we conducted and by the demonstration of the role of Locus 3 in fish infections thanks to in vivo experiments. We are pleased that the reviewer recognizes the significance of our identification of three functional groups driving host specificity in GBS. Additionally, we appreciate the acknowledgment of our findings that reiterate the association of scpB acquisition with human interactions and the Lac.2 operon with cattle on a much wider scale and with more robust and comprehensive methods than what has been previously done. We are excited to contribute novel insights into GBS evolution, particularly with the discovery of Locus 3 and its role in fish host adaptation, and we believe these findings will pave the way for further research in this area. Thank you for your thorough and constructive review.

Part II – Major Issues: Key Experiments Required for Acceptance
Please use this section to detail the key new experiments or modifications of existing experiments that should be absolutely required to validate study conclusions.

Generally, there should be no more than 3 such required experiments or major modifications for a "Major Revision" recommendation. If more than 3 experiments are necessary to validate the study conclusions, then you are encouraged to recommend "Reject".

Reviewer #1: (No Response)

The authors note the absence of suggestions from reviewer #1 under the "major issues" section.

Reviewer #2: (No Response)

The authors note the absence of suggestions from reviewer #2 under the "major issues" section.

Part III – Minor Issues: Editorial and Data Presentation Modifications
Please use this section for editorial suggestions as well as relatively minor modifications of existing data that would enhance clarity.

Reviewer #1: (No Response)

The authors note the absence of suggestions from reviewer #1 under the "minor issues" section.

Reviewer #2:

- Assuming mobile genetic element but 'MGE' should be defined in Ln 30. Spelled out in Ln 208
    - *Thank you for noticing, the text has not been updated (line 31 and line 213).*

- Explanation of how 80% was selected as the cutoff for host generalist v host specialists would be helpful.
    - *The explanation was present in the supplementary appendix (section A.3) and in the legend of Fig.1, but thanks to the reviewer's comment we decided to also add it to the main text to improve clarity (lines 145-148).*

- Fig 2B could benefit from grouping the CGs by host generalist, host-adapted, and host restricted rather than numberical order across the X-axis
    - *This was changed, thank you for the suggestion as we also agree this improved readability of the figure.*

- Ln 168-169: Are the authors able to indicate where SL61 falls on Fig. 2C?
    - *Unfortunately, this is not possible as genomes from SL61 are scattered across the main cluster: genomes of SL61 vary in size from 1.99 Mbp to 2.46 Mpb (all values can be retrieved from the Supplementary table S1), with a mean and median of 2.14 Mb, and therefore they do not form a separate cluster as SL552. This is because the pseudogenisation of SL61 is described in its capsular genes (Almeida et al., 2016), and it did not lead to the kind of genome reduction as observed in SL552. Fig.2 in line 168-169 referred uniquely to SL552, and the text has now been changed for clarity (now line 173).*

- Could the authors add labels or comment on the enriched human-associated unitigs that fall between 0.50 and 0.75 in the reference genome position in Fig 4A, especially the peak ~0.6.
    - *Thank you for pointing that. The small peak in correspondence to position 0.67 in the human-associated unitigs corresponds to some phage tail proteins. In this figure (4A and 4B) we only labelled peaks of genes that were detected as significant by both*

GWAS methods (pyseer and scoary), which was not the case for the phage tail genes in the human phenotype. This information has been added to the legend of Fig 4 to clarify why that peak is not annotated.

- It is difficult to determine where the supplemental data citations are referencing. The tables are not cited in the text. In Table S2, what do the colors indicate? Are there corresponding legends to the supplemental data?
    - *We apologise for the lack of clarity on the supplementary files. Citations were already present for "Supplementary appendix file" in section 2 (Materials and methods), and for "Supplementary tables S2-S4" (scoary results) within the Supplementary appendix file (section B3). However, to improve clarity, citations were added in the main text for: Supplementary table S1 (dataset and results) in section 2.1 (line 66), and in in section 3.4 for Supplementary table S2 (scoary results for human adaptation) (line 225), Supplementary table S3 (scoary results for bovine adaptation) (line 242), and Supplementary table S4 (scoary results for fish adaptation) (line 259). The colours within tables S2-S4 were added as a gradient to improve readability (higher to lower values) but they have now been removed.*

- The key for Fig. 6A says 'Viet Nam'
    - *Changed to "Vietnam"*

- Ln 304-308: Are the colors representing Brazil and Vietnam inverted in this figure? The text cites that the emergence of the CG283 strains in Brazil occurred around 2015 but the figure looks as if this is the Vietnam strain emergence. And vice versa for the 2003 scpB loss that, according to the figure, appears to be isolated from strains in Brazil, the text cites Vietnam.
    - *When interpreting the tree, it can be appreciated how the Brazilian clade (dark brown) clusters within the Vietnamese (dark violet) isolates, with dark violet isolates flanking the brown ones on both sides. The first ST283 isolates available from Brazil date back to 2015, and indeed emergence likely happened before (~2012 based on BEAST estimates). The text has been updated for clarity (lines 313-318).*
      Please bear in mind that the annotations on the external strips (e.g., country of origin) refer to the tips of the tree, which are the available genomes, not to the internal nodes. The loss of scpB in 2003, which is marked with an arrow, refers to an internal node, not to the tips, which means that it refers to the common ancestor of the entire scpB-negative clade (which comprises isolates from Vietnam, Thailand and Brazil). This information uniquely concerns the estimated genomic content of the common ancestral strain (i.e. scpB-negative), and in no way it gives information on its country of origin.
